# Supplementary figures and images for: Proteomics, physiological, and biochemical analysis of cross tolerance mechanisms in response to heat and water stresses in soybean
Source: PLoS One. 2020 Jun 5;15(6):e0233905. doi: 10.1371/journal.pone.0233905 (PMC7274410; doi:10.1371/journal.pone.0233905)

## Slide 1
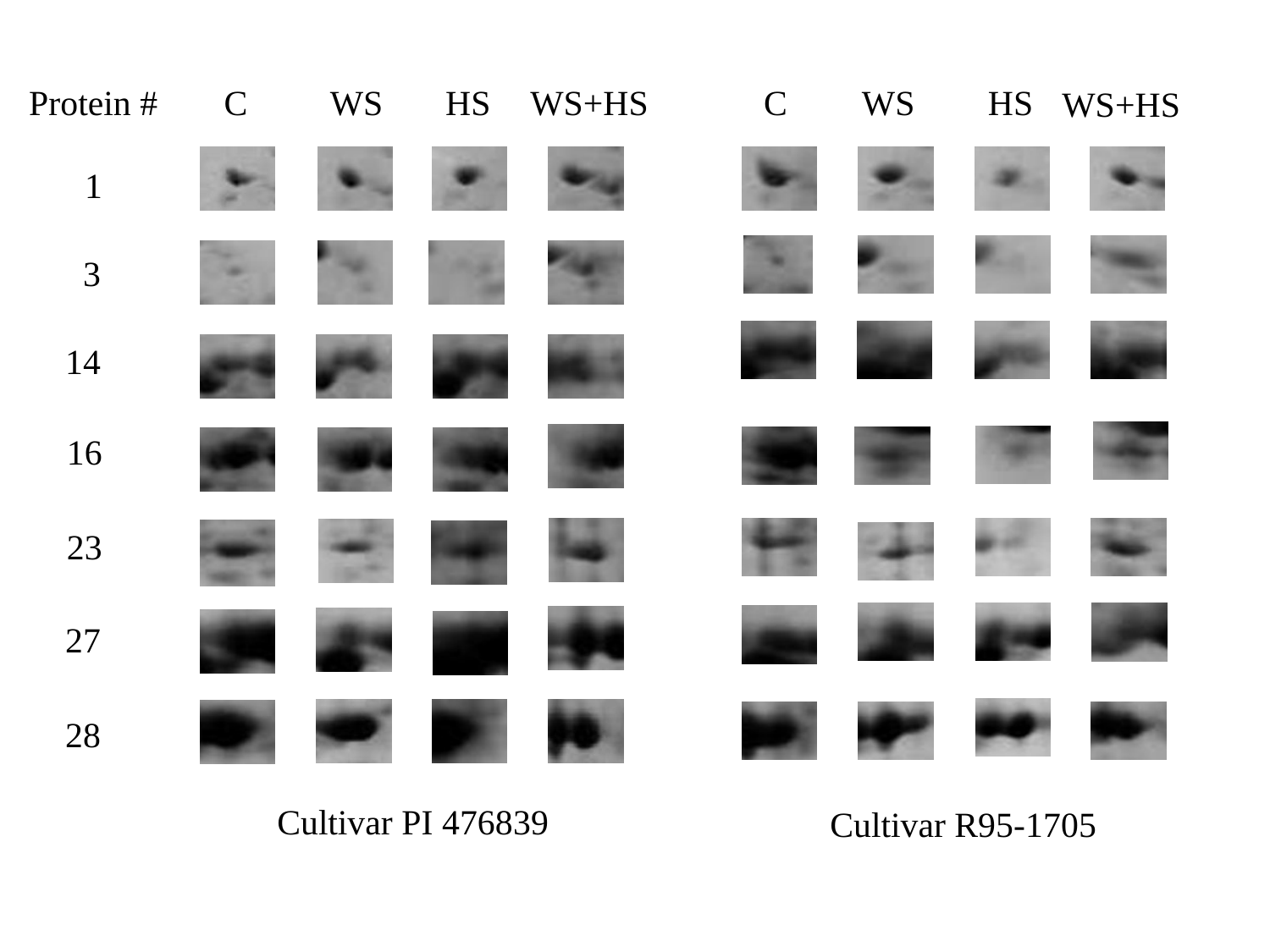

Protein #
C
WS
HS
WS+HS
C
WS
HS
WS+HS
 1
 3
14
16
23
27
28
Cultivar PI 476839
Cultivar R95-1705

Supplement: S1 Fig — (PPT) [file pone.0233905.s001.ppt]

Graphical Abstract:

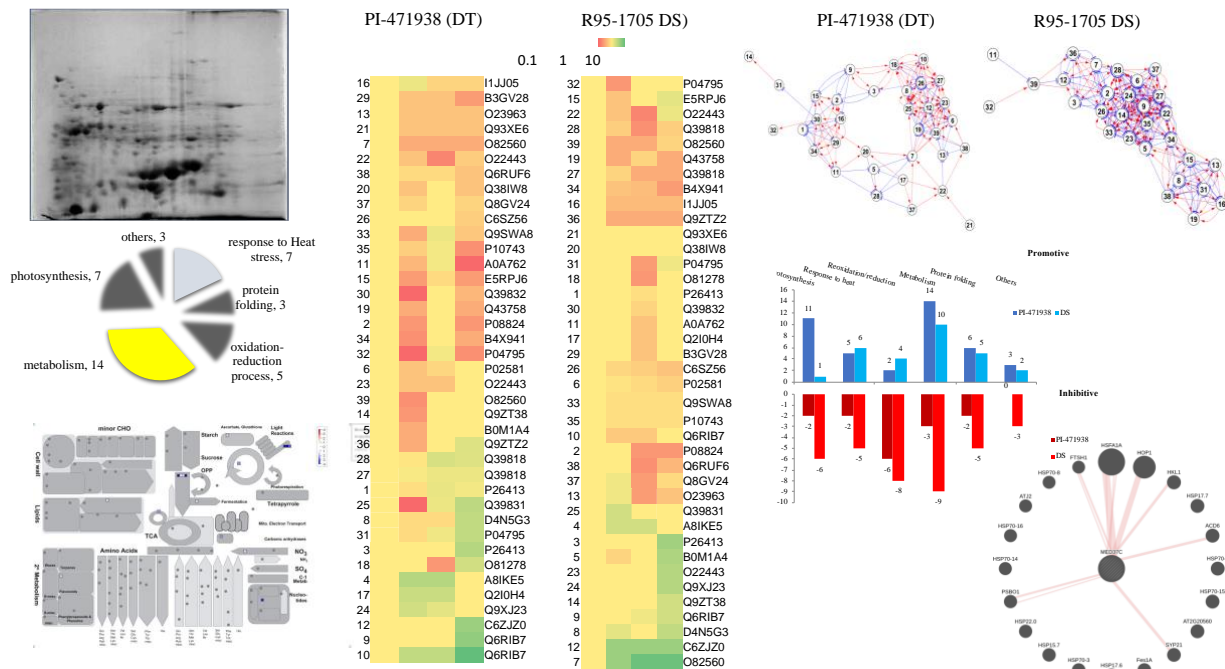

Supplement: S1 Graphical Abstract — (PDF) [file pone.0233905.s010.pdf]
