# Supplementary material for: Proteomics, physiological, and biochemical analysis of cross tolerance mechanisms in response to heat and water stresses in soybean
Source: PLoS One. 2020 Jun 5;15(6):e0233905. doi: 10.1371/journal.pone.0233905 (PMC7274410; doi:10.1371/journal.pone.0233905)
Supplement: S2 Fig — (PPTX) [file pone.0233905.s002.pptx]

## Slide 1
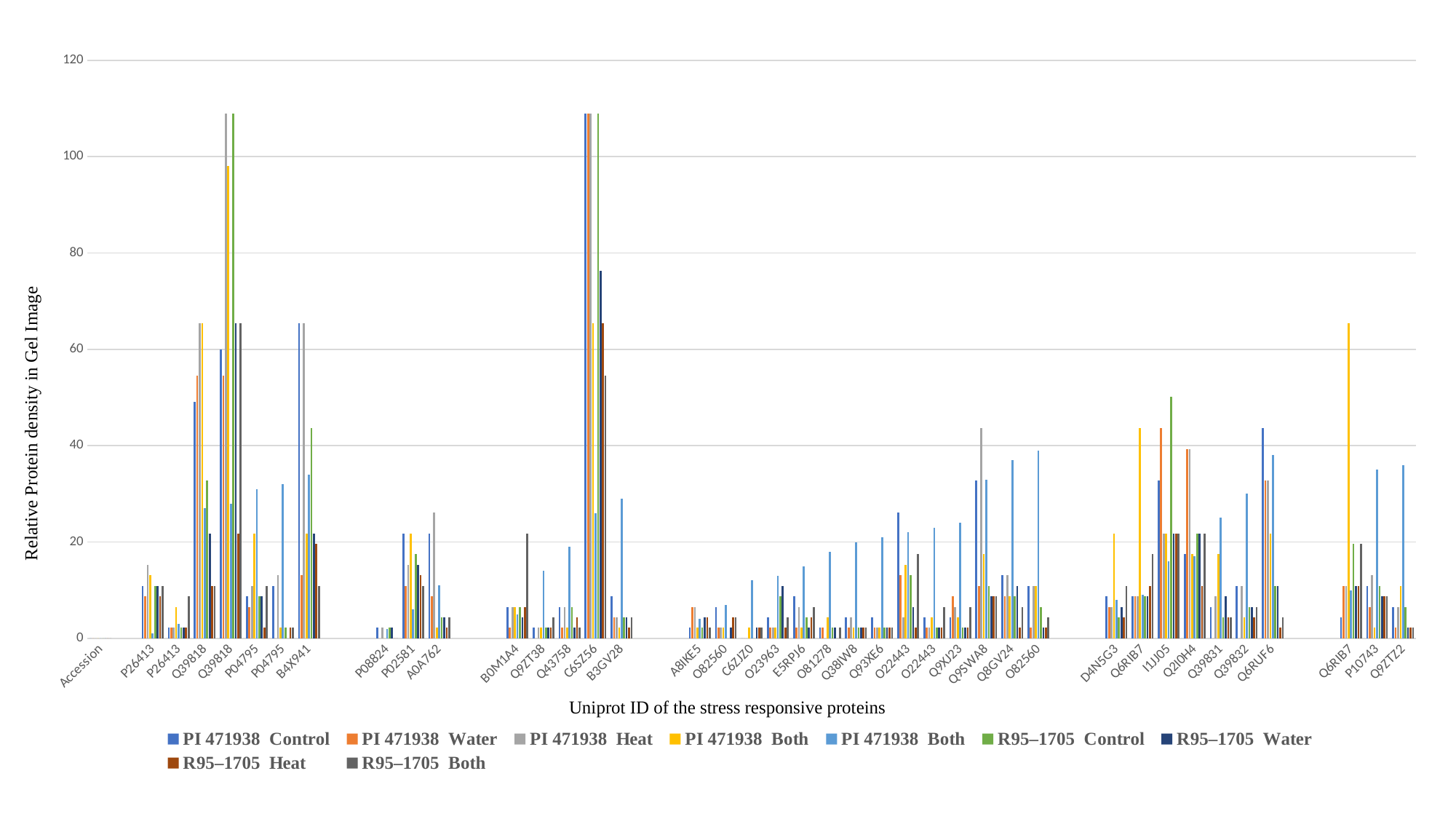

### Chart
| Category | PI 471938 | PI 471938 | PI 471938 | PI 471938 | PI 471938 | R95–1705 | R95–1705 | R95–1705 | R95–1705 |
|---|---|---|---|---|---|---|---|---|---|
| Accession | 0.0 | 0.0 | 0.0 | 0.0 | 0.0 | 0.0 | 0.0 | 0.0 | 0.0 |
| | None | None | None | None | None | None | None | None | None |
| P26413 | 10.9 | 8.72 | 15.26 | 13.08 | 1.0 | 10.9 | 10.9 | 8.72 | 10.9 |
| P26413 | 2.18 | 2.18 | 2.18 | 6.540000000000001 | 3.0 | 2.18 | 2.18 | 2.18 | 8.72 |
| Q39818 | 49.050000000000004 | 54.50000000000001 | 65.4 | 65.4 | 27.0 | 32.7 | 21.8 | 10.9 | 10.9 |
| Q39818 | 59.95 | 54.50000000000001 | 109.00000000000001 | 98.10000000000001 | 28.0 | 109.00000000000001 | 65.4 | 21.8 | 65.4 |
| P04795 | 8.72 | 6.540000000000001 | 10.9 | 21.8 | 31.0 | 8.72 | 8.72 | 2.18 | 10.9 |
| P04795 | 10.9 | 0.0 | 13.080000000000002 | 2.18 | 32.0 | 2.18 | 0.0 | 2.18 | 2.18 |
| B4X941 | 65.4 | 13.080000000000002 | 65.4 | 21.8 | 34.0 | 43.6 | 21.8 | 19.62 | 10.9 |
| | None | None | None | None | None | None | None | None | None |
| | None | None | None | None | None | None | None | None | None |
| P08824 | 2.18 | 0.0 | 2.18 | 0.0 | 2.0 | 2.18 | 2.18 | 0.0 | 0.0 |
| P02581 | 21.8 | 10.9 | 15.260000000000002 | 21.8 | 6.0 | 17.44 | 15.260000000000002 | 13.080000000000002 | 10.9 |
| A0A762 | 21.8 | 8.72 | 26.160000000000004 | 2.18 | 11.0 | 4.36 | 4.36 | 2.18 | 4.36 |
| | None | None | None | None | None | None | None | None | None |
| | None | None | None | None | None | None | None | None | None |
| B0M1A4 | 6.540000000000001 | 2.18 | 6.540000000000001 | 6.540000000000001 | 5.0 | 6.540000000000001 | 4.36 | 6.540000000000001 | 21.8 |
| Q9ZT38 | 2.18 | 0.0 | 2.18 | 2.18 | 14.0 | 2.18 | 2.18 | 2.18 | 4.36 |
| Q43758 | 6.540000000000001 | 2.18 | 6.540000000000001 | 2.18 | 19.0 | 6.540000000000001 | 2.18 | 4.36 | 2.18 |
| C6SZ56 | 109.00000000000001 | 109.00000000000001 | 109.00000000000001 | 65.4 | 26.0 | 109.00000000000001 | 76.30000000000001 | 65.4 | 54.50000000000001 |
| B3GV28 | 8.72 | 4.36 | 4.36 | 2.18 | 29.0 | 4.36 | 4.36 | 2.18 | 4.36 |
| | None | None | None | None | None | None | None | None | None |
| | None | None | None | None | None | None | None | None | None |
| A8IKE5 | 2.18 | 6.540000000000001 | 6.540000000000001 | 2.18 | 4.0 | 2.18 | 4.36 | 4.36 | 2.18 |
| O82560 | 6.540000000000001 | 2.18 | 2.18 | 2.18 | 7.0 | 0.0 | 2.18 | 4.36 | 4.36 |
| C6ZJZ0 | 0.0 | 0.0 | 0.0 | 2.18 | 12.0 | 0.0 | 2.18 | 2.18 | 2.18 |
| O23963 | 4.36 | 2.18 | 2.18 | 2.18 | 13.0 | 8.72 | 10.9 | 2.18 | 4.36 |
| E5RPJ6 | 8.72 | 2.18 | 6.540000000000001 | 2.18 | 15.0 | 4.36 | 2.18 | 4.36 | 6.540000000000001 |
| O81278 | 2.18 | 2.18 | 0.0 | 4.36 | 18.0 | 2.18 | 2.18 | 0.0 | 2.18 |
| Q38IW8 | 4.36 | 2.18 | 4.36 | 2.18 | 20.0 | 2.18 | 2.18 | 2.18 | 2.18 |
| Q93XE6 | 4.36 | 2.18 | 2.18 | 2.18 | 21.0 | 2.18 | 2.18 | 2.18 | 2.18 |
| O22443 | 26.160000000000004 | 13.080000000000002 | 4.36 | 15.260000000000002 | 22.0 | 13.080000000000002 | 6.540000000000001 | 2.18 | 17.44 |
| O22443 | 4.36 | 2.18 | 2.18 | 4.36 | 23.0 | 2.18 | 2.18 | 2.18 | 6.540000000000001 |
| Q9XJ23 | 4.36 | 8.72 | 6.540000000000001 | 4.36 | 24.0 | 2.18 | 2.18 | 2.18 | 6.540000000000001 |
| Q9SWA8 | 32.7 | 10.9 | 43.6 | 17.44 | 33.0 | 10.9 | 8.72 | 8.72 | 8.72 |
| Q8GV24 | 13.080000000000002 | 8.72 | 13.080000000000002 | 8.72 | 37.0 | 8.72 | 10.9 | 2.18 | 6.540000000000001 |
| O82560 | 10.9 | 2.18 | 10.9 | 10.9 | 39.0 | 6.54 | 2.18 | 2.18 | 4.36 |
| | None | None | None | None | None | None | None | None | None |
| | None | None | None | None | None | None | None | None | None |
| D4N5G3 | 8.72 | 6.540000000000001 | 6.540000000000001 | 21.8 | 8.0 | 4.36 | 6.540000000000001 | 4.36 | 10.9 |
| Q6RIB7 | 8.72 | 8.72 | 8.72 | 43.6 | 9.0 | 8.72 | 8.72 | 10.9 | 17.44 |
| I1JJ05 | 32.7 | 43.6 | 21.8 | 21.8 | 16.0 | 50.14 | 21.8 | 21.8 | 21.8 |
| Q2I0H4 | 17.44 | 39.24 | 39.24 | 17.44 | 17.0 | 21.8 | 21.8 | 10.9 | 21.8 |
| Q39831 | 6.540000000000001 | 0.0 | 8.72 | 17.44 | 25.0 | 4.36 | 8.72 | 4.36 | 4.36 |
| Q39832 | 10.9 | 0.0 | 10.9 | 4.36 | 30.0 | 6.540000000000001 | 6.540000000000001 | 4.36 | 6.540000000000001 |
| Q6RUF6 | 43.6 | 32.7 | 32.7 | 21.8 | 38.0 | 10.9 | 10.9 | 2.18 | 4.36 |
| | None | None | None | None | None | None | None | None | None |
| | None | None | None | None | None | None | None | None | None |
| Q6RIB7 | 4.36 | 10.9 | 10.9 | 65.4 | 10.0 | 19.62 | 10.9 | 10.9 | 19.62 |
| P10743 | 10.9 | 6.540000000000001 | 13.080000000000002 | 2.18 | 35.0 | 10.9 | 8.72 | 8.72 | 8.72 |
| Q9ZTZ2 | 6.540000000000001 | 2.18 | 6.540000000000001 | 10.9 | 36.0 | 6.540000000000001 | 2.18 | 2.18 | 2.18 |Relative Protein density in Gel Image
Uniprot ID of the stress responsive proteins
