# Supplementary material for: Proteomics, physiological, and biochemical analysis of cross tolerance mechanisms in response to heat and water stresses in soybean
Source: PLoS One. 2020 Jun 5;15(6):e0233905. doi: 10.1371/journal.pone.0233905 (PMC7274410; doi:10.1371/journal.pone.0233905)
Supplement: S3 Fig — (PPTX) [file pone.0233905.s003.pptx]

## Slide 1
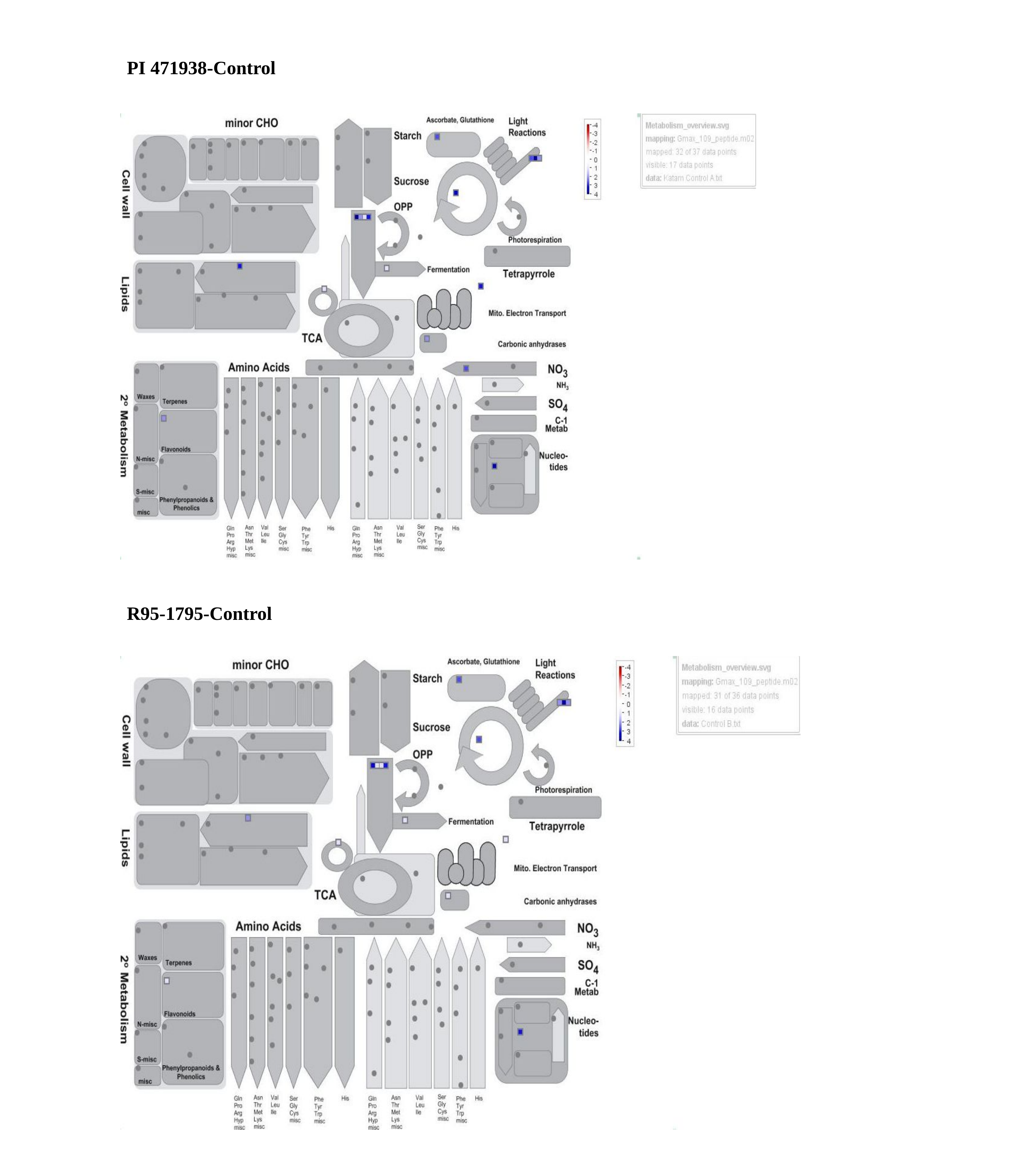

PI 471938-Control
R95-1795-Control

## Slide 2
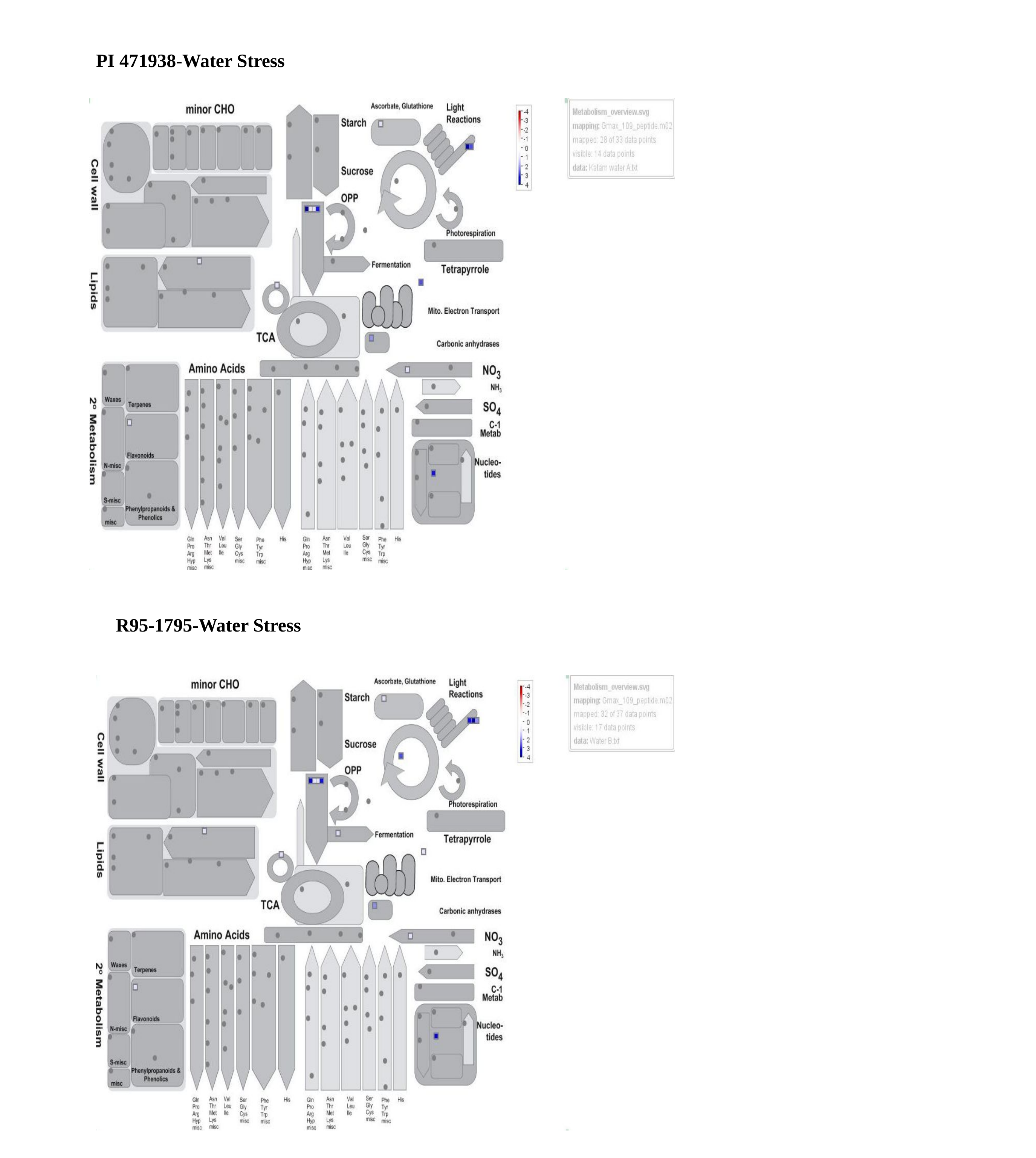

PI 471938-Water Stress
R95-1795-Water Stress

## Slide 3
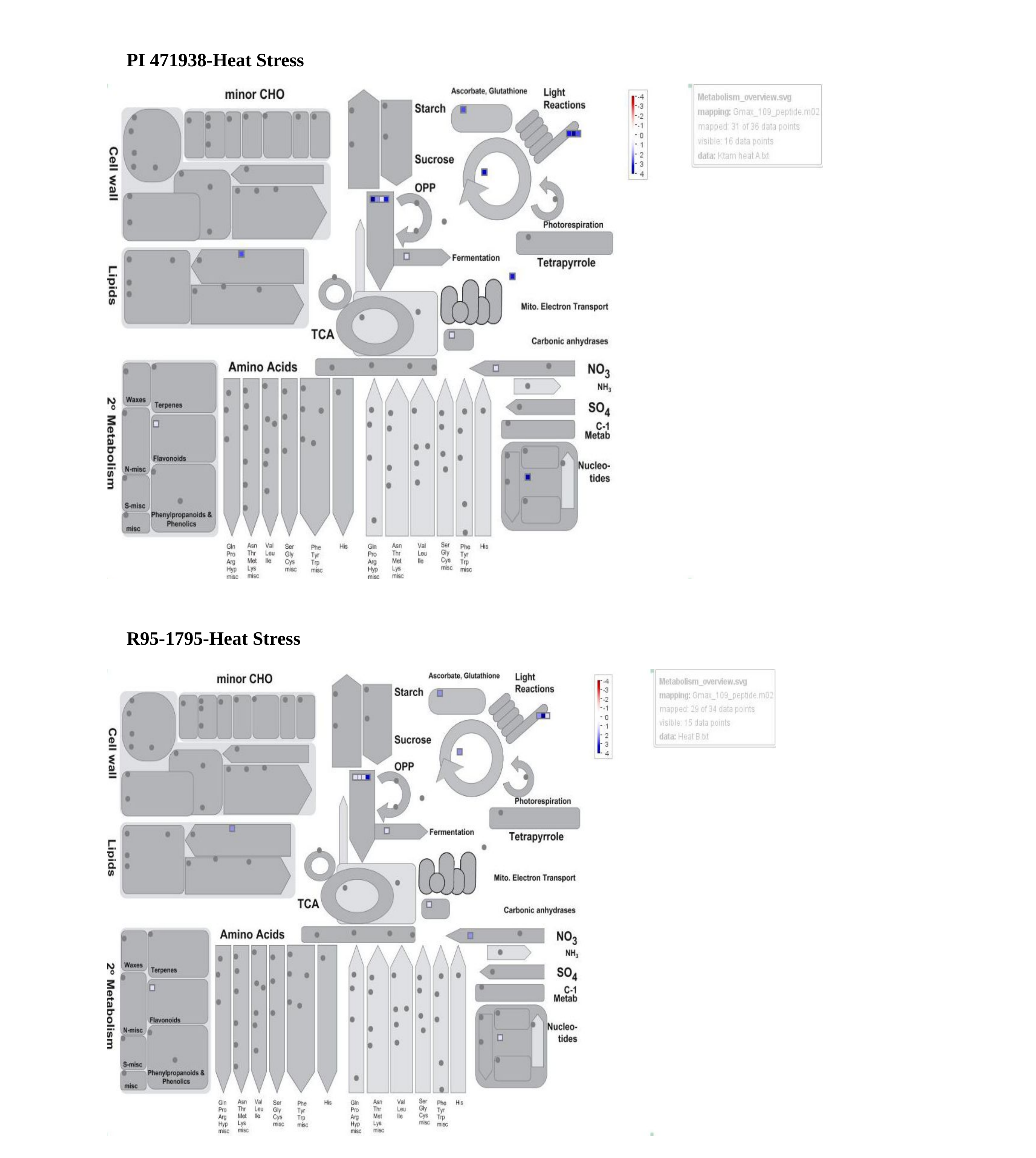

PI 471938-Heat Stress
R95-1795-Heat Stress
