# Supplementary material for: Proteomics, physiological, and biochemical analysis of cross tolerance mechanisms in response to heat and water stresses in soybean
Source: PLoS One. 2020 Jun 5;15(6):e0233905. doi: 10.1371/journal.pone.0233905 (PMC7274410; doi:10.1371/journal.pone.0233905)
Supplement: S1 Table — (PDF) [file pone.0233905.s004.pdf]

Supplementary Table I- List of primers used for real-time RT-qPCR

**Uniprot Identifier**	**Protein Name**	**Gene name**	**Forward Primer Sequences -->**	**Reverse Primer Sequences ←**
Q7XZI9	Actin	NM_114519.2	TGCGTCTTGATCTTGCAGGT	GAAGCACTTCCTGTGGACGA
Q9SXT2	Ascorbate peroxide	AB024991.1	ATGCCACCAAAGGTTCTGAC	AGGCCTTCCTTCTCACCATT
Q9XF98	Calreticulin	AF134733.1	ACTGGTAGCCTCTACAGCGA	TGCAGCTTCTTCTCCAGCTC
Q93XE6	Chalcone flavone isomerase	Glyma 20g38560	GTGCGTGTGTGTCTTACAGC	ATCATGGTCTCCAAGACCGC
O82560	Glutamine synthetase	Glyma 14g39430	ATGACAGGCTACCAT	TTTGCAATCGCAAGT
P26413	Heat shock Protein 70	Glyma 17g08020	TAGCGTTGATTTTGGCCTGC	GCGTCCAATTGCGTCAAACT
Q9SZH2	Peroxidase	BX829145.1	ATGGCTCGATCCTCGTGAAC	ACGCACGTGACACTAACACA
Q9FE12	Peroxiredoxin	AJ288896.1	TTGCCAGGGCTTCGGTAATT	TTGCTTCAAAGGTCAGGCCA
C6ZJZO	Serine hydroxyl methyltransferase 5	Glyma 18g150000	TTGAGAGATGGGCAAGAGCA	ATGTGACGCGCACTACCATT
C6SZ56	Superoxide dismutase	Glyma 19g42890	GCTGTCTGTCGCTTTTGACG	CTGGAGCTAATGGTCAGCAGT

All primers were designed using NCBI database and the Primer3Plus.
