# Supplementary material for: Proteomics, physiological, and biochemical analysis of cross tolerance mechanisms in response to heat and water stresses in soybean
Source: PLoS One. 2020 Jun 5;15(6):e0233905. doi: 10.1371/journal.pone.0233905 (PMC7274410; doi:10.1371/journal.pone.0233905)
Supplement: S2 Table — (PDF) [file pone.0233905.s005.pdf]

Supplementary Table II: Two-Dimensional Electrophoresis Gel Reproducibility

**Sl. No.**	**Treatment**	**Cultivar PI- 471938**	**% Change**	**Cultivar R95-1705**	**% Change**
1	Control	224		212	
2	Water Stress (WS)	167	Decrease (25)	198	Decrease (6.6)
3	Heat Stress (HS)	242	Increase (8)	142	Decrease (33)
4	Water and Heat Stress (WS+HS)	201	Decrease (10)	190	Decrease (10)

Average number of protein spots in three replicates. Protein Spots having high quality score as assigned by PD Quest V 8.2.1
