# Supplementary material for: Proteomics, physiological, and biochemical analysis of cross tolerance mechanisms in response to heat and water stresses in soybean
Source: PLoS One. 2020 Jun 5;15(6):e0233905. doi: 10.1371/journal.pone.0233905 (PMC7274410; doi:10.1371/journal.pone.0233905)
Supplement: S3 Table — (PDF) [file pone.0233905.s006.pdf]

Supplementary Table III: Promotive and Inhibitive Effect of Stress Responsive Proteins

Cultivar PI 471938			Cultivar R95-1705		
Source Protein #	Sink Protein #		Source Protein #	Sink Protein #	
	Promotive	Inhibitive		Promotive	Inhibitive
1		2,11,15,16,29,30,34	2	26,28,36	3,7,12
2		3,9	3	7,12	2,6,26,28,36
3	9,18	2	5	9,14,22,23,24	2,6,26,27,33,34,35
5	28	7	6	2,26,27,28,33,35	5,9,14,22,23,24
6	8,23,25,27,39	12,19,26	7	3,12	2,6,26,28,36
7	6,12,23,25,39	19	8	5,15,31	13,16,19,34,38
8	10,12,18,23,25,27	26	9	5,14,22,23,24	2,6,26,27,28,33,35
9	3,18	2,15,16	11		39
10	8,12,18,25,27	26	12	3,7	2,26,28,36
11	20	5,28	13	16,19,34,38	8,15,31
12	6,8,10,23,25,27,39	19,26	14	5,9,22,23,24	2,6,27,28,33,34,35,37
13	19	6,12,23,39	15	5,8,22,23,31	13,16,19,34,38
15	2,16	1,9	16	13,19,38	8,15,31
16	2,15,29,34,30	1	19	13,16,38	8,15,31
17	20,22,37	5,28	22	5,9,14,15,23,24	2,6,27,33,34,35
18	3,8,9,10,25,27	26	23	5,9,14,22,24	2,6,26,27,33,34,35
19	26	6,8,12,23,25,27,39	24	5,9,14,22,23	2,6,26,27,28,33,34,34,37

20	7	28	26	2,6,27,28,33,35	3,5,7,9,14,22,23,24
21	22		27	2,6,28,33,34,35,37	5,9,14,22,23,24
22	7,38,	13	28	2,6,27,35,37	9,14,24,22,23
23	6,8,10,12,25,27,39	19,26	31	8	13,16,19,38
25	8,10,12,18,23,27	26	32	39	
26	27	3,8,10,12,18,25	33	2,6,26,27,28,35	5,7,9,14,22,23,24
27	8,10,12,18,25	26	34	2,13,27,38	5,9,8,15,22,23,31
28		7	35	2,6,22,27,28,33,34,37	5,9,14,23,24
29	2,11,15,16,30,34	1	36	2,26,28	3,7,12
30	11,20,32,34	31	37	2,27,34,35	9,14,22,24
31	14		38	13,16,19,34	5,8,15,31
34	2,11,15,16,29,30	1	39	2,30	12
37	7,22	28			
38	6,23,39	13			
39	12,17,23,25	19,26			
**Total**					
**32**	**95**	**61**	**29**	**113**	**139**

Source/sink protein number/s reflect the protein spot numbers on the 2DE gel.
