# Supplementary material for: Proteomics, physiological, and biochemical analysis of cross tolerance mechanisms in response to heat and water stresses in soybean
Source: PLoS One. 2020 Jun 5;15(6):e0233905. doi: 10.1371/journal.pone.0233905 (PMC7274410; doi:10.1371/journal.pone.0233905)
Supplement: S4 Table — (PDF) [file pone.0233905.s007.pdf]

Supplementary Table IV: Promotive and Inhibitive Effect of Stress Responsive Proteins to combined stresses

Cultivar PI 471938

Source		Sink			
**Protein #**	**Biological Function**		**Biological Function of Promotive interaction**		**Biological Function of inhibitive interaction**
3	Response to heat	9	Glycolytic process	2	Protein refolding
		18	Carbohydrate metabolic process, Malat metabolic process,Tricarboxylicacid process		
31	Response to heat	14	Oxidation, reduction		
34	Response to heat	2	protein refolding	1	Stress-related, Protein refolding
		11	Signal transduction		
		15	TCA		
		16	Photosynthesis		
		29	Cell Redox hemostasis		
		30	Photosynthesis, carbon fixation,photorespiration		
5	Oxidation-reduction	28	Stress-related	7	Glutamine biosynthetic process, Metabolism
7	Metabolism	6	Signal transduction	19	Redox, cellular response to oxidative stress
		12, 23, 39	Metabolism		
		25	Photosynthesis,light harvesting		
12	Metabolism	6	Signal transduction	19	Redox, cellular response to oxidative stress

		8	Photosynthesis	26	Redox
		10			
		23, 39	Metabolism		
		25	Photosynthesis,light harvesting		
		27	Stress-related		
15	Metabolism	2	Protein refolding	1	Stress-related, Protein refolding
		16	Photosynthesis	9	Glycolytic process
22	Metabolism	7	Glutamine biosynthetic process, Metabolism	13	Metabolism
		38	Photosynthesis,glycolytic process		
23		6	Signal transduction	19	Redox, cellular response to oxidative stress
		8	Photosynthesis	26	Redox
		10			
		12, 39	Metabolism		
		25	Photosynthesis,light harvesting		
		27	Stress-related		
8	Photosynthesis	10		26	Redox
		12, 23	Metabolism		
		18	Carbohydrate metabolic process, Malat metabolic process,Tricarboxylicacid process		
		25	Photosynthesis,light harvesting		
		27	Stress-related		
9	Photosynthesis	3	Stress-related, Protein refolding	2	Protein refolding

	18	Carbohydrate metabolic process, Malat metabolic process,Tricarboxylicacid process	15	TCA
			16	Photosynthesis
**Total 11**	**41**		**15**	
