# Supplementary material for: Proteomics, physiological, and biochemical analysis of cross tolerance mechanisms in response to heat and water stresses in soybean
Source: PLoS One. 2020 Jun 5;15(6):e0233905. doi: 10.1371/journal.pone.0233905 (PMC7274410; doi:10.1371/journal.pone.0233905)
Supplement: S5 Table — (DOCX) [file pone.0233905.s008.docx]

|  | | | | | |
| --- | --- | --- | --- | --- | --- |
| **Supplementary Table V: Supplementary Table IV: Promotive and Inhibitive Effect of Stress Responsive Proteins showing high abundance in response to combined Water and Heat stresses** | | | | | |
|  |  |  | **Cultivar R95–1705** |  |  |
| **Source** | | **Sink** | | | |
|  |  |  |  |  |  |
| **Protein #** | **Biological Function** | **Promotive** | **Biological Function** | **Inhibitive** | **Biological Function** |
| 3 | Response to heat | 7,12 | Metabolism | 2 | Protein refolding |
|  |  |  |  | 6 | Signal transduction |
|  |  |  |  | 26 | redox |
|  |  |  |  | 28 | Stress-related |
|  |  |  |  | 36 | Seed development |
|  |  |  |  |  |  |
| 27 | Response to heat | 2 | Protein refolding | 5 | Redox, responsive to hydrogen peroxide |
|  |  | 6 | Signal transduction | 9 | Glycolytic process |
|  |  | 28,34 | Response to heat | 14 | Oxidation, reduction |
|  |  | 33,37 | Metabolism | 22 | Hydrogen peroxide catabolic process, Metabolism |
|  |  | 35 | Seed storage | 23,24 | Metabolism |
|  |  |  |  |  |  |
| 28 | Response to heat | 2 | Protein refolding | 9 | Glycolytic process |
|  |  | 6 | Signal transduction | 14 | Oxidation, reduction |
|  |  | 27 | Stress-related | 22 | Hydrogen peroxide catabolic process, Metabolism |
|  |  | 35 | Acid and other phosphatases | 23,24 | Metabolism |
|  |  | 37 | Metabolism |  |  |
|  |  |  |  |  |  |
| 31 | Response to heat | 8 | Photosynthesis | 13 | Metabolism |
|  |  |  |  | 16 | Photosynthesis |
|  |  |  |  | 19 | Redox, cellular response to oxidative stress |
|  |  |  |  | 38 | Photosynthesis, glycolytic process |
|  |  |  |  |  |  |
| 12 | Metabolism | 3 | Stress-related, Protein refolding | 2 | Protein refolding |
|  |  | 7 | Glutamine biosynthetic process, Metabolism | 26 | redox |
|  |  |  |  | 28 | Stress-related |
|  |  |  |  | 36 | Seed development |
|  |  |  |  |  |  |
| 8 | Photosynthesis | 5 | Redox, responsive to hydrogen peroxide | 13 | Metabolism |
|  |  | 15 | TCA | 16 | Photosynthesis |
|  |  | 31 | Stress-related, Protein complex oligomerization, Protein folding, Response to heat | 19 | Redox, cellular response to oxidative stress |
|  |  |  |  | 34 | Stress-related |
|  |  |  |  | 38 | Photosynthesis, glycolytic process |
|  |  |  |  |  |  |
| 9 | Photosynthesis | 5 | Redox, responsive to hydrogen peroxide | 2 | Protein refolding |
|  |  | 14 | Oxidation, reduction | 6 | Signal transduction |
|  |  | 22 | Hydrogen peroxide catabolic process, Metabolism | 26 | Redox |
|  |  | 23,24 | Metabolism | 27,28 | Stress-related |
|  |  |  |  | 33 | Metabolism |
|  |  |  |  | 35 | Acid and other phosphatases |
|  |  |  |  |  |  |
| 36 | Others | 2 | Protein refolding | NA |  |
|  |  | 26 | redox |  |  |
|  |  | 28 | Stress-related |  |  |
|  | Subtotal | 28 |  | 36 |  |
|  |  |  |  |  |  |
