# Supplementary material for: Proteomics, physiological, and biochemical analysis of cross tolerance mechanisms in response to heat and water stresses in soybean
Source: PLoS One. 2020 Jun 5;15(6):e0233905. doi: 10.1371/journal.pone.0233905 (PMC7274410; doi:10.1371/journal.pone.0233905)

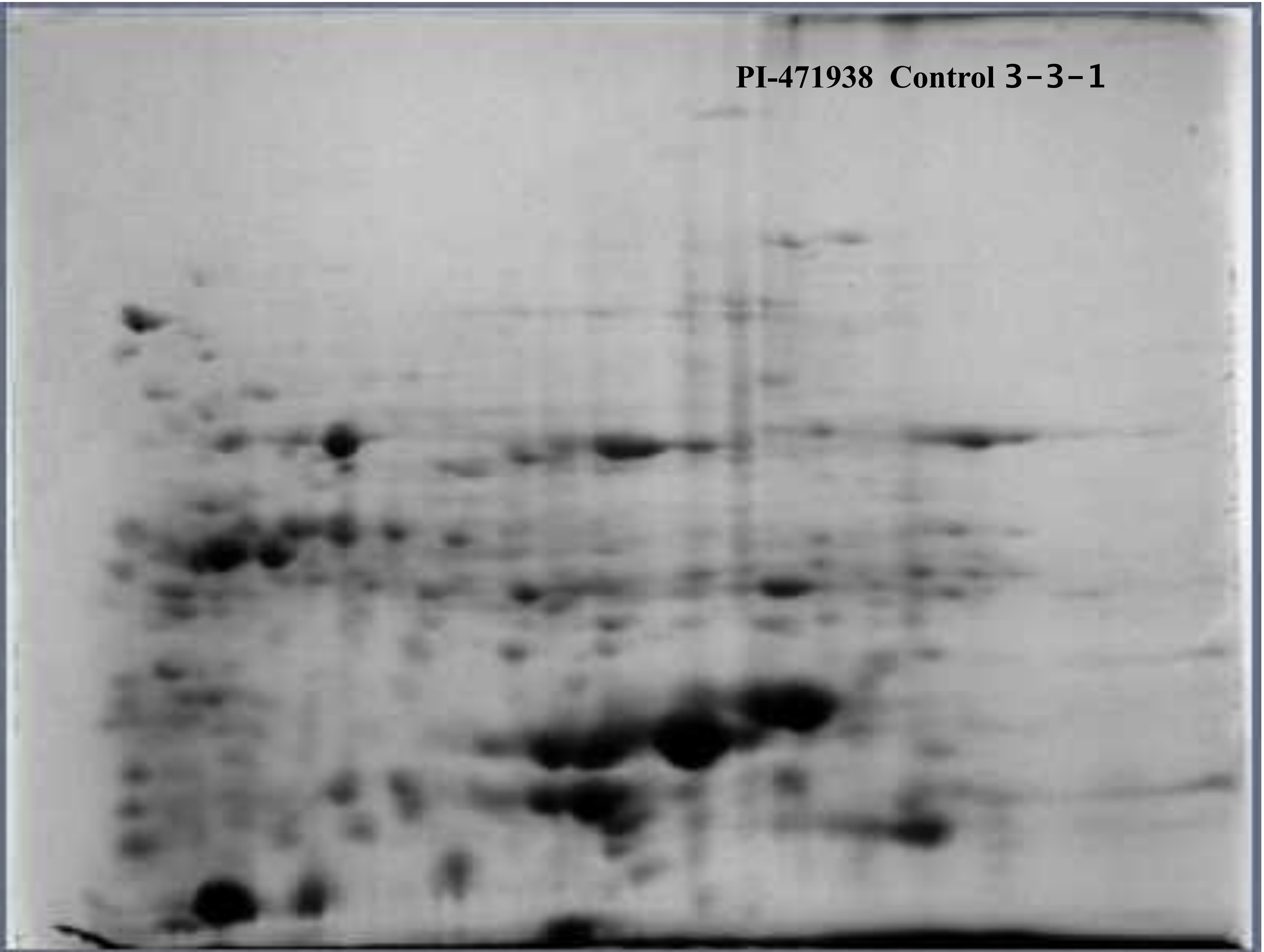
PI-471938 Control 3-3-1

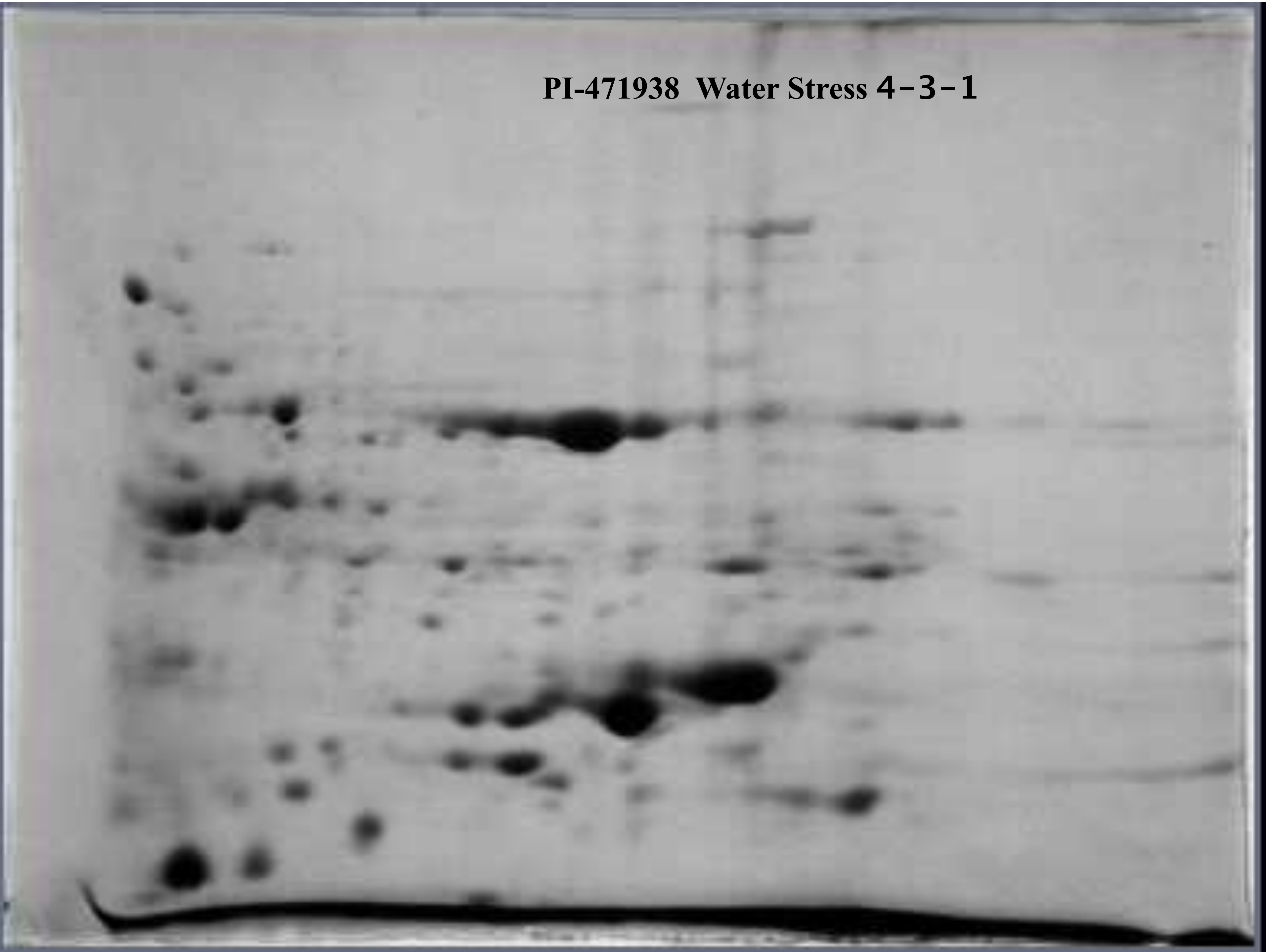
PI-471938 Water Stress 4-3-1

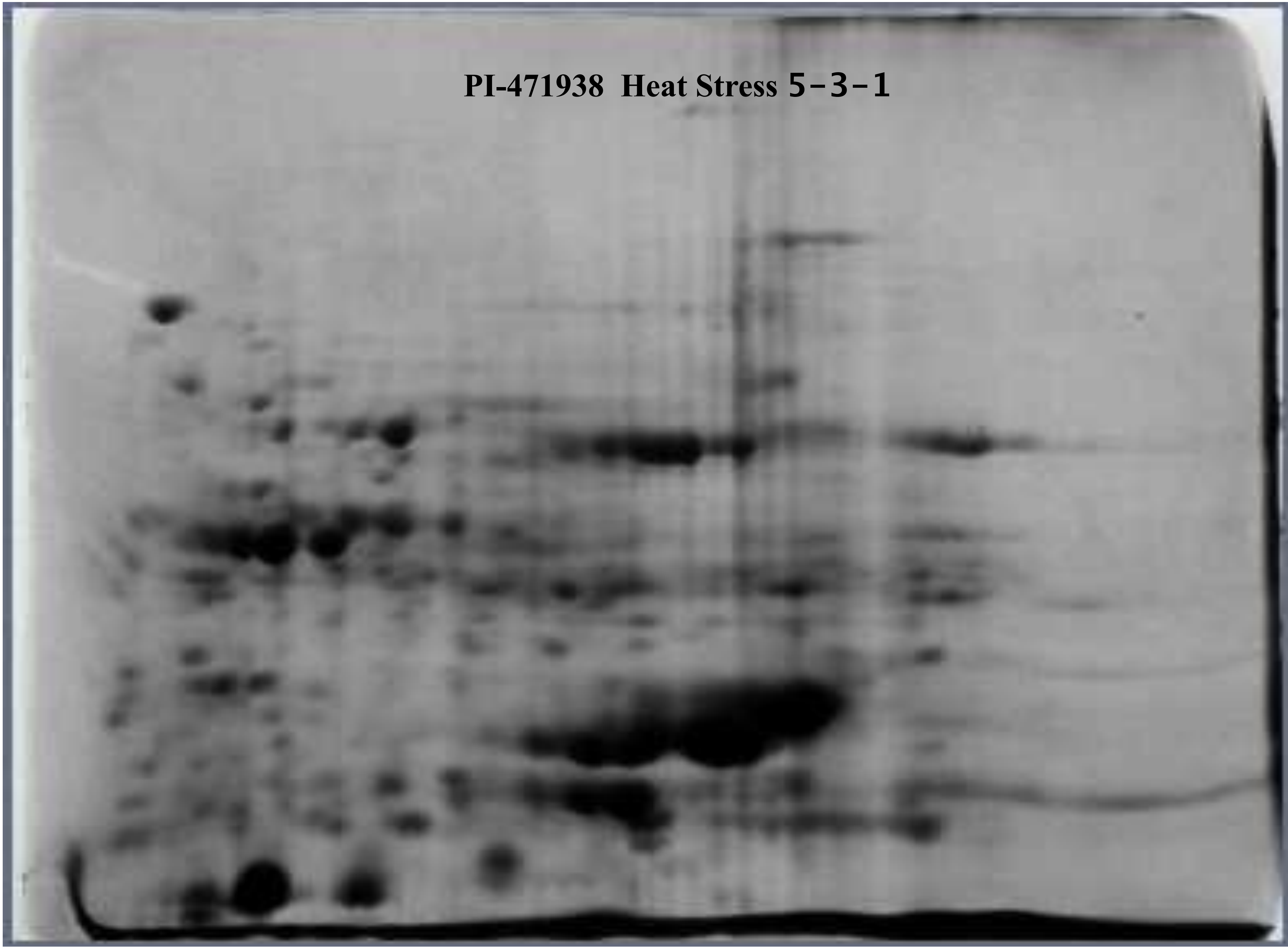
PI-471938 Heat Stress 5-3-1

PI-471938 Water and Heat Stress 6-3-1

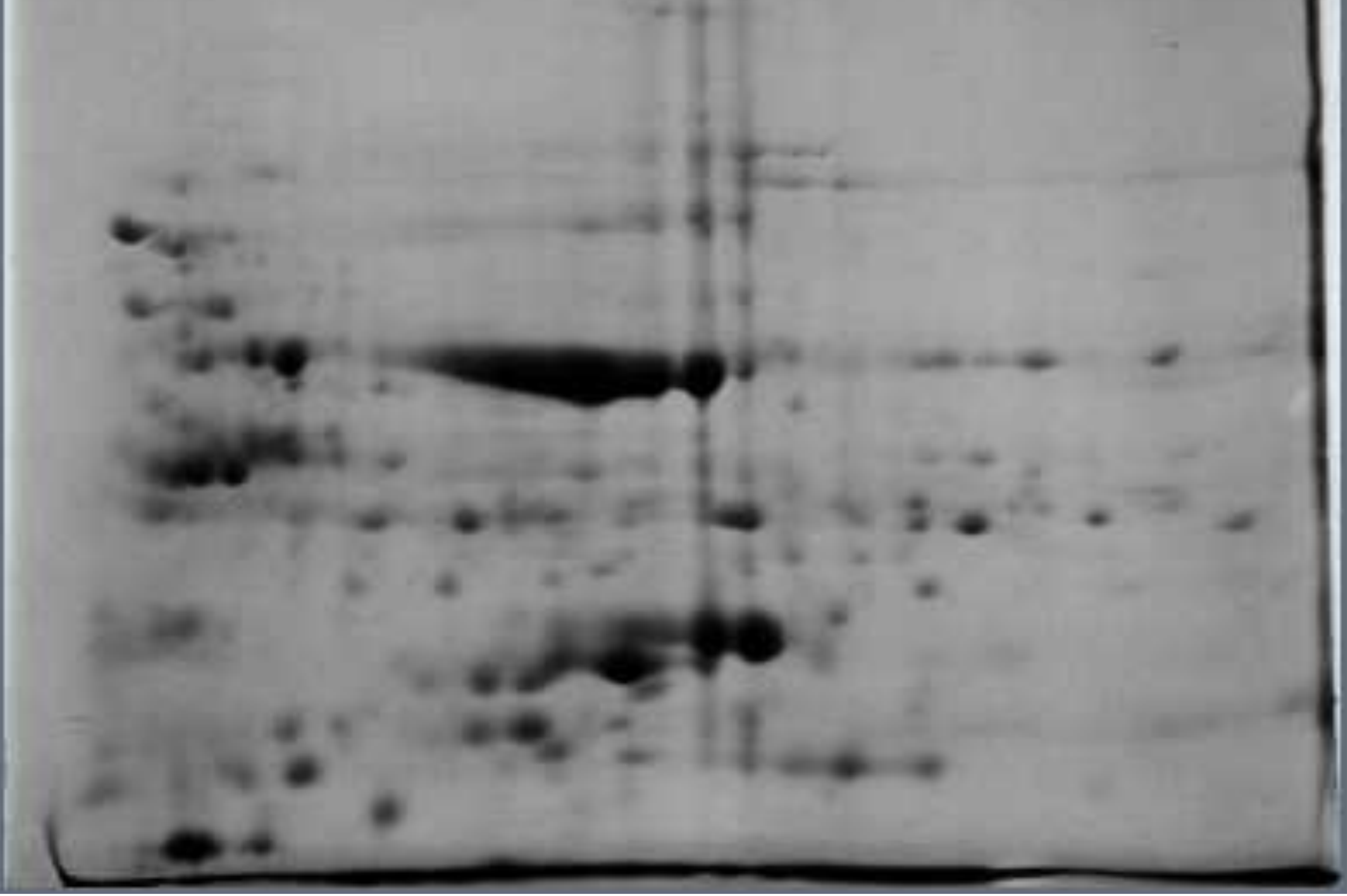

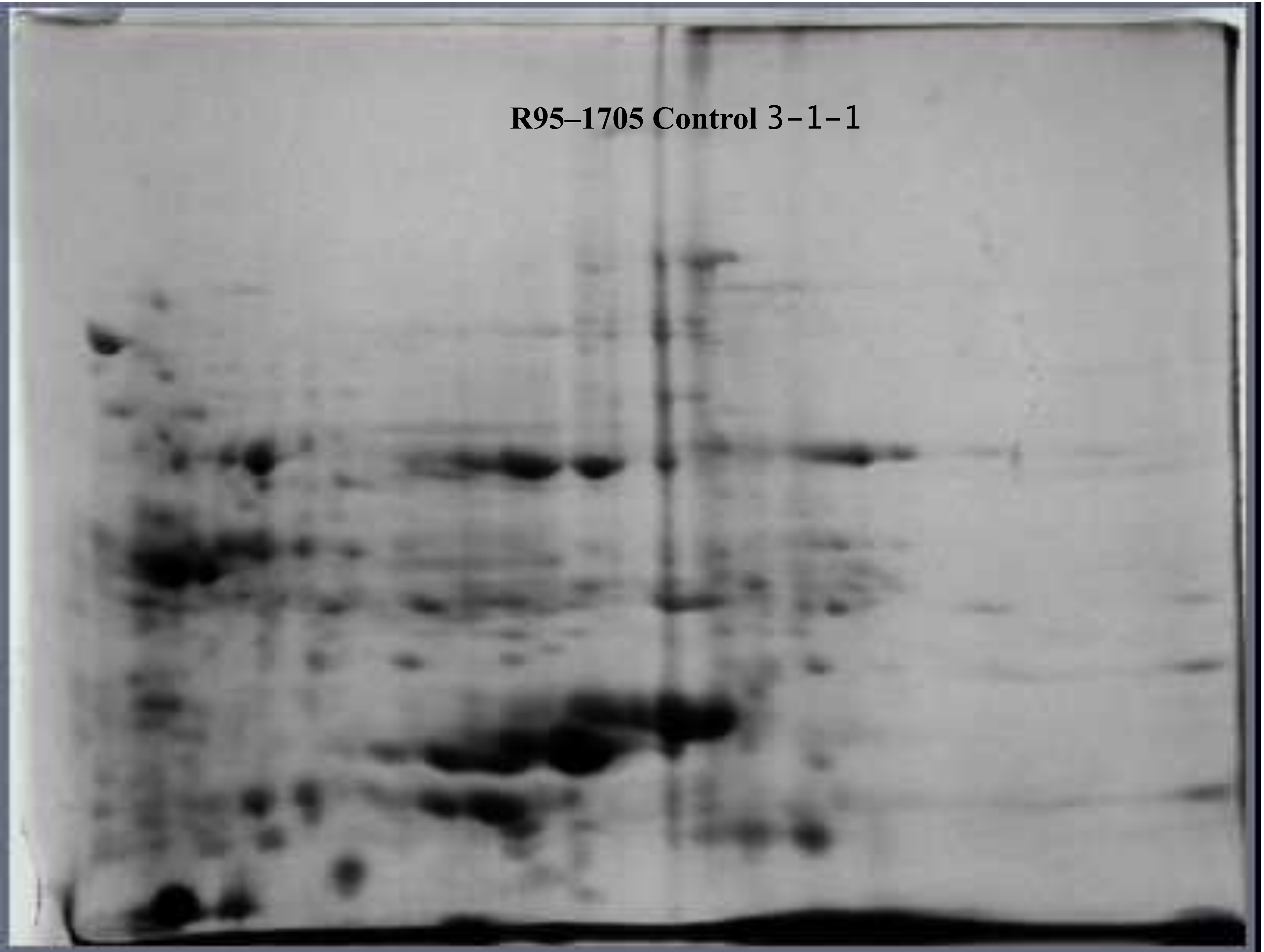
R95–1705 Control 3-1-1

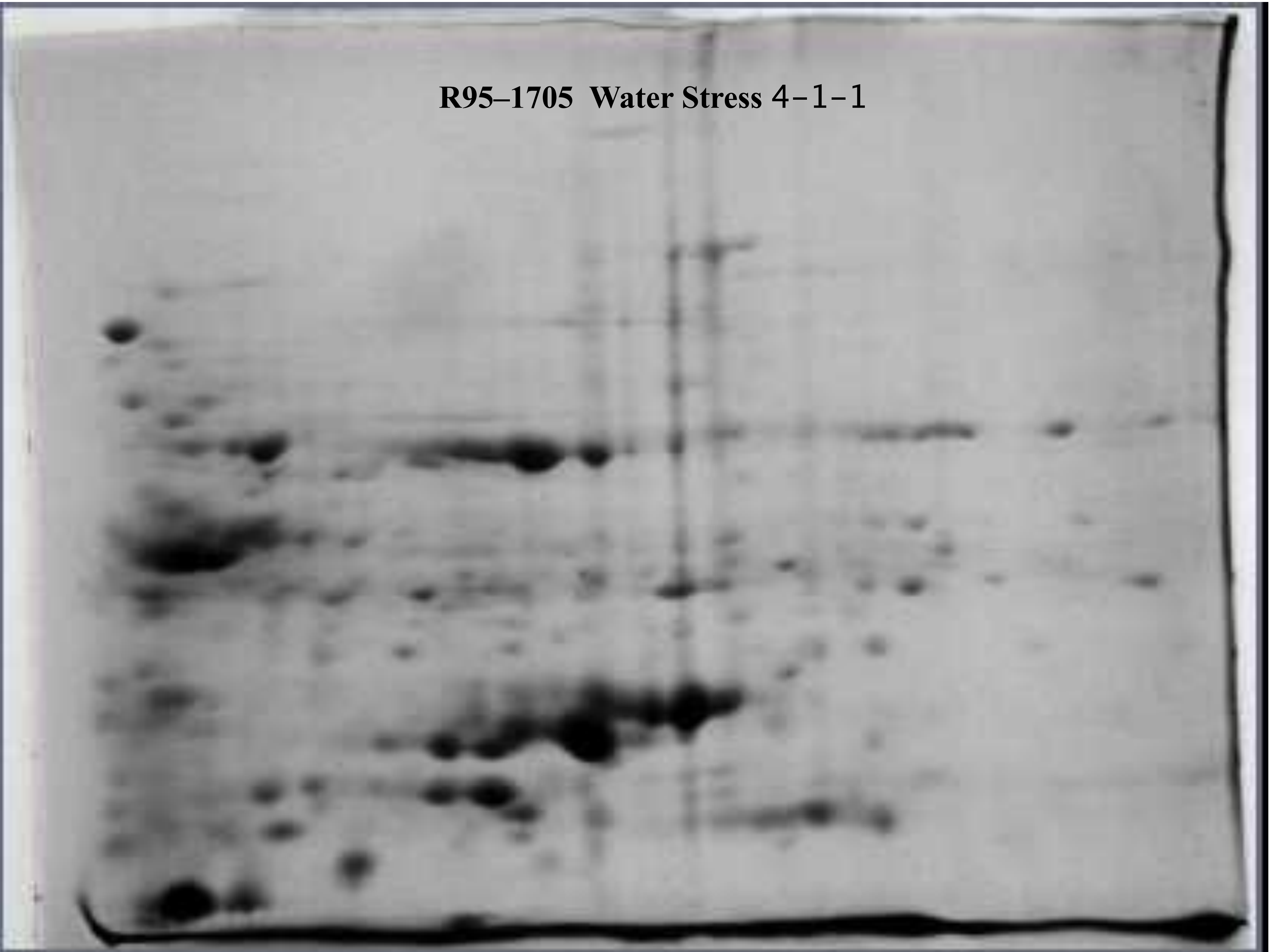
R95–1705 Water Stress 4-1-1

R95–1705 Heat Stress 5-1-1

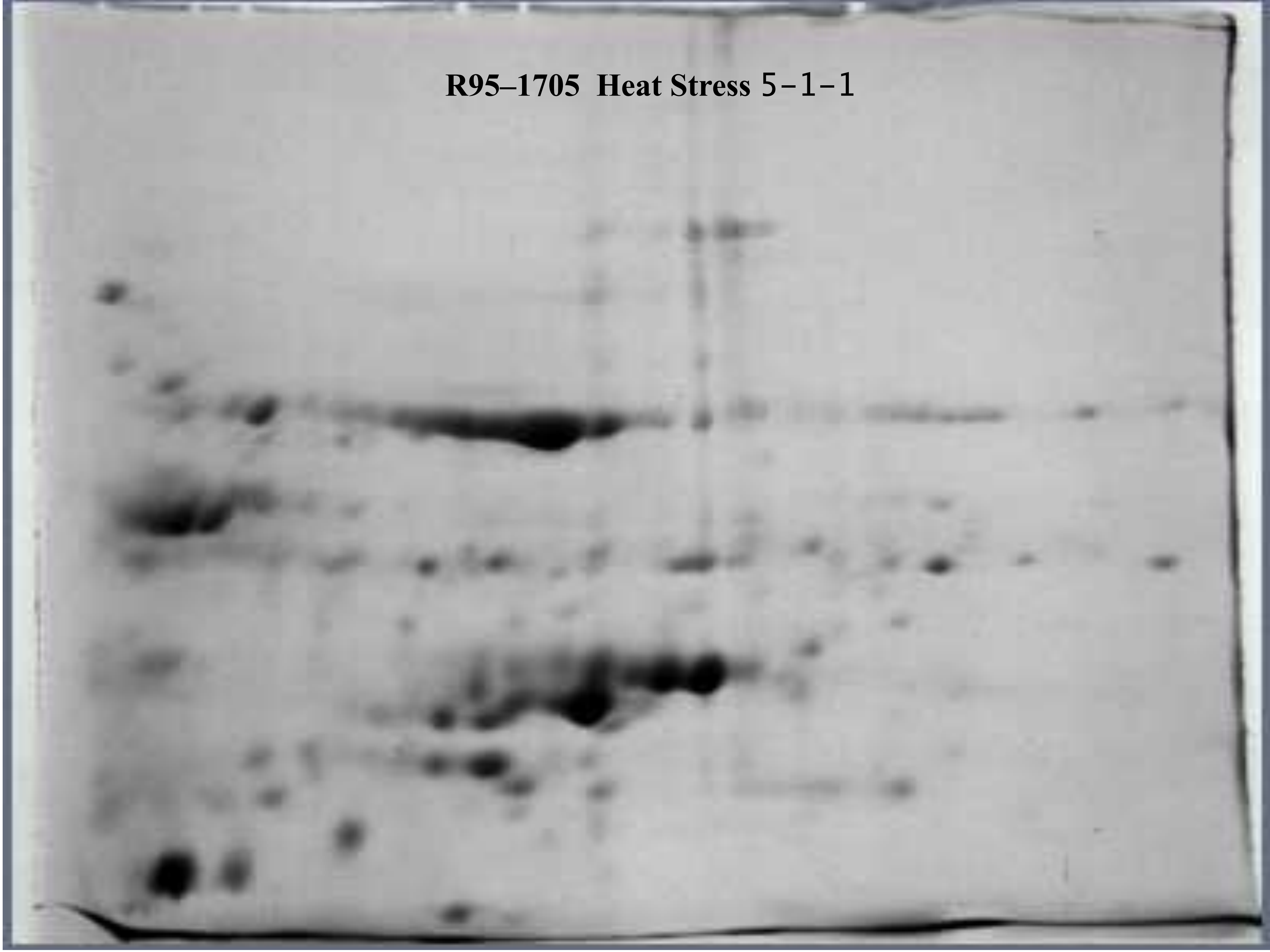

R95–1705 Water and Heat Stress 6-1-1

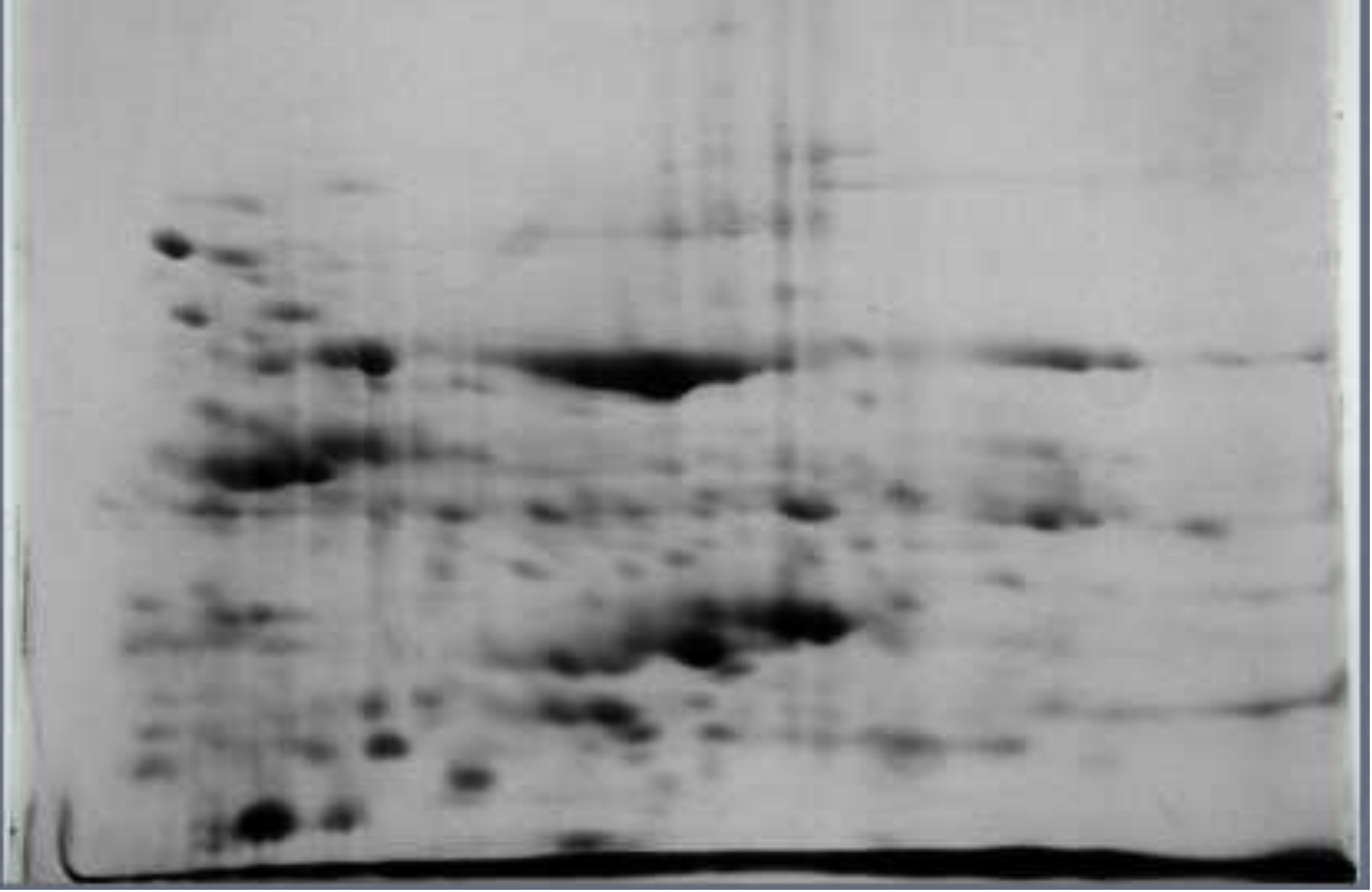

Supplement: S1 Data — (PDF) [file pone.0233905.s009.pdf]
