# Supplementary material for: Proteomics, physiological, and biochemical analysis of cross tolerance mechanisms in response to heat and water stresses in soybean
Source: PLoS One. 2020 Jun 5;15(6):e0233905. doi: 10.1371/journal.pone.0233905 (PMC7274410; doi:10.1371/journal.pone.0233905)
Supplement: Attachment — Submitted filename: Response to Reviewers.docx [file pone.0233905.s011.docx]

05/15/2020

Dear Academic Editor,

Thank you very much for considering the manuscript suitable for publication in PLoS One journal. We included the following files; Rebuttal, Manuscript, and Revised Manuscript With Track Changes for further review and consideration. Please note tht our responses are highlighted in BLUE.

We look forward to receiving your decision on this manuscript.

Kind regards,

Ramesh Katam


When submitting your revision, we need you to address these additional requirements:

1. Please ensure that your manuscript meets PLOS ONE's style requirements, including those for file naming. The PLOS ONE style templates can be found at <http://www.plosone.org/attachments/PLOSOne_formatting_sample_main_body.pdf> and <http://www.plosone.org/attachments/PLOSOne_formatting_sample_title_authors_affiliations.pdf>

Response: Entire manuscript was revised to meet PLOS ONE’s style requirements.

2. Please include your tables as part of your main manuscript and remove the individual files. Please note that supplementary tables should remain as separate "supporting information" files

Response: Tables were included as part of main manuscript and removed the individual files. Supplementary tables were included as “supporting information’ files.

3. PLOS ONE now requires that authors provide the original uncropped and unadjusted images underlying all blot or gel results reported in a submission’s figures or Supporting Information files. This policy and the journal’s other requirements for blot/gel reporting and figure preparation are described in detail at <https://journals.plos.org/plosone/s/figures#loc-blot-and-gel-reporting-requirements> and <https://journals.plos.org/plosone/s/figures#loc-preparing-figures-from-image-files>. When you submit your revised manuscript, please ensure that your figures adhere fully to these guidelines and provide the original underlying images for all blot or gel data reported in your submission. See the following link for instructions on providing the original image data: <https://journals.plos.org/plosone/s/figures#loc-original-images-for-blots-and-gels>.

In your cover letter, please note whether your blot/gel image data are in Supporting Information or posted at a public data repository, provide the repository URL if relevant, and provide specific details as to which raw blot/gel images, if any, are not available. Email us at [plosone@plos.org](mailto:plosone@plos.org) if you have any questions.

Response: Original gels uncropped were included as Supporting Information.

4. Please upload a copy of Supporting Information Table V which you refer to in your text on page 23.
[Note: HTML markup is below. Please do not edit.]

Response: Supporting Information Table V is included.

Reviewers' comments:

Reviewer's Responses to Questions

**Comments to the Author**

1. Is the manuscript technically sound, and do the data support the conclusions?

The manuscript must describe a technically sound piece of scientific research with data that supports the conclusions. Experiments must have been conducted rigorously, with appropriate controls, replication, and sample sizes. The conclusions must be drawn appropriately based on the data presented.

Reviewer #1: Yes

Response: Thank you very much for the comments on the quality of the manuscript data.

2. Has the statistical analysis been performed appropriately and rigorously?

Reviewer #1: No

Response: Statistical analysis has been performed for all the figures and for the data of the manuscript and revised as needed.

3. Have the authors made all data underlying the findings in their manuscript fully available?

The [PLOS Data policy](https://nam03.safelinks.protection.outlook.com/?url=http%3A%2F%2Fwww.plosone.org%2Fstatic%2Fpolicies.action%23sharing&data=02%7C01%7C%7Cbded8b049d264523e4b208d78dd0ed45%7C2526db3cd5034dfea0e60c41a20b52d2%7C0%7C1%7C637133800799451958&sdata=t4gNpuBjEwIXlS86F37hoVXZf2qHpBRKdCjJJPc6bmM%3D&reserved=0) requires authors to make all data underlying the findings described in their manuscript fully available without restriction, with rare exception (please refer to the Data Availability Statement in the manuscript PDF file). The data should be provided as part of the manuscript or its supporting information, or deposited to a public repository. For example, in addition to summary statistics, the data points behind means, medians and variance measures should be available. If there are restrictions on publicly sharing data—e.g. participant privacy or use of data from a third party—those must be specified.

Reviewer #1: Yes

Response: Thank you for your comment.

4. Is the manuscript presented in an intelligible fashion and written in standard English?
PLOS ONE does not copyedit accepted manuscripts, so the language in submitted articles must be clear, correct, and unambiguous. Any typographical or grammatical errors should be corrected at revision, so please note any specific errors here.

Reviewer #1: Yes

Response: Thank you for your comment.

5. Review Comments to the Author

Please use the space provided to explain your answers to the questions above. You may also include additional comments for the author, including concerns about dual publication, research ethics, or publication ethics. (Please upload your review as an attachment if it exceeds 20,000 characters)

Reviewer #1: Remarks:
> The Authors tested two soybean cultivars. One of the cultivars is characterized as „slow wilting and high yielding” and the second one as “high in protein concentration and moderate yield potential’. I am not sure if they differ from each other as regards the phenotype connected with the studied stresses. I suggest giving information on their susceptibility to the studied stresses.

Response: These are newly developed cultivars and have not been fully studied for their response to drought. However, the PI genotype has been tested for heat-tolerance and showed those tolerance traits. Additional information on these cultivars are provided in the revised manuscript.

> Besides, the Authors use different names for the tested two cultivars. I don't understand why it is so. It is absolutely necessary to sort it out. The cultivar PI 471938 is also called: Cultivar#2; PI; 471938; PI 373819; PI-371938; DT, whilst the cultivar R95 – 1795 is also named: Cultivar#1; R-95; R95-1705; R95; R 95-1705; DS.

Response: The cultivar names were revised and are consistent in the entire manuscript.

Material and Methods
> line 147: Physiological measurements - how many replications were used for the analysis?

Response: we used 6 replications for all physiological measurements and 10 replications for total biomass. Accordingly, the materials and methods section was updated.

> line 162: Statistical analysis – this subsection should be transferred to the end of the section Material and Methods

Response: The Statistical analysis subsection was transferred to the end of section Materials and Methods.

> line 222: Quantitative Real-Time Polymerase Chain Reaction (RT-q PCR) Analysis: What plant material and in what amount was used for RNA isolation? What method was used for RNA isolation? How was the reverse transcription performed? Please, give the PCR parameters (annealing temperature and time). Method for identifying the RT-qPCR products; method for evaluating the quantity of transcript. What was the reference gene, if any? Were there negative controls? Was the effectiveness of RT-PCR amplification assessed using standard gel electrophoresis? The expression of which genes was studied? Please, name these genes.

Response: Thank you for your comment. Revised and included the details about plant material, quantity, RNA isolation method, RT PCR parameters, reference gene, list of genes quantified, and the method of product validation.

> line 228: Enzyme assay: No description of how the samples were prepared for determination, and what plant material was used for that purpose. How much plant material was used for determination? How many biological replicates were made? Lack of statistics.

Response: Detailed description of sample preparation, plant material used, including biological replicates was explained in methods section. Figures associated with enzyme assay was prepared using statistics.

Results
> Abbreviations, when used for the first time in Material and Methods, should be explained. Pnet, Tr, TBM, Fv’/Fm’. How was TBM determined? – no description in Material and Methods.

Response: Thanks. We have explained in the materials and methods section and also in the Table.

> lines 248-250, and Table 1 – please check the font.

Response: Fonts were checked and revised.

> line 275; correlation coefficient: please check it

Response: Checked and corrected.

> line 308: “mixed response” please explain what it means

Response: Revised.

> line 316: “protein 7 and 12 …..were not present” – it is not a correct interpretation, this would mean that in the control no proteins undergo expression, while the level of the tested proteins shows an increase or decrease in relation to the control point (see the scale) which was 1.

Response: Revised and deleted the statement.

> line 344: Supplementary Table V is not present in the manuscript

Response: Supplementary Table V is provided.

> line 358: Figure 9b is not present in the manuscript

Response: Figure 9 b which is related to cultivar R95-1705 is included.

> line 371: “significantly higher” – in order to conclude that something is significantly higher, ANOVA should have been performed.

Response: Performed ANOVA and verified for significant differences and the data is provided as supplementary document.

Figure and Table Legends:
Figure titles are insufficient. There is a lack of figure legends, so in consequence the interpretation of the presented results is complicated or even impossible.

Response: Figure titles are described in detail with the legends for better visual expression.

Please, check the title of Figure 6, in the title of Figure 7 – “what does A:” mean?

Response: Revised and given the cultivar names.

Figure 9 title: “transcript expression’ is not a proper term: I suggest to use “transcript accumulation” or “transcript level”

Response: Revised to “transcript level”

Figures
Figure 1: needs to be improved, please check the names of the cultivars.

Response: Figure 1 is improved and corrected the cultivar names.

Figure 2: Please, explain in the legend what 3-1-1 etc. mean; please, check the names of the cultivars

Response: Revised the names of cultivars and the treatments.

Figure 3: is not clear, please explain what Figure 3b expresses

Response: Information provided.

Figure 5: needs to be improved

Response: Improved Figure 5

Figure 6: Please, give information about the treatment under the column for both the cultivars. Please sign the A) and B) parts to the figure. Please name the part of the figure with the numbers on the left and right sides; the clusters are redrawn to improve the visibility.

Response: The clusters are redrawn to improve the visibility. The numbers were added on left and right side of the cluster diagrams. Figure part a and b were assigned and labeled the cultivar name. Figure was numbered on left and right sides with the numbers and uniport IDs.

Figure 7: It should be improved. It is improper and unnecessary to introduce additional determinations for cultivars in that part of the manuscript. The figure is not divided into a, b and c, the references to which are in the text and the legend. Please, explain what the numbers on the graph mean. The lower part of the figure is a chart which has unfortunately been constructed in a way which makes it impossible to see all the data

Response: The figure quality is improved. The figure is divided into a, b and c in reference to the information in the text. The numbers in the graph indicate number of proteins related to the biological functions affected. The graph was re-constructed to show more details.

Figure 9: There is a lack of the part connected with the cultivar R95-1705. Please, check the name of the proteins – please, see also Supplementary Table I. Relative mRNA abundance – no description of the axis. Protein – no description of the axis. In general, a lack of statistics. No explanation of what the figure is about. Proteins – how ‘protein abundance’ was calculated?

Response: Figure 9 (a & b) is about the relation to transcript level and corresponding protein relative abundance. The protein names were revised and also corrected in Supplementary Table I. X and Y axis for mRNA and protein were described in the table. Protein abundance was calculated using PD Quest software (Bio Rad) which uses the algorithm of relative protein spot density. In this method, the raw quantity of each spot in a member gel is divided by the total intensity value of all the pixels in the image.

Figure 10. Lack of statistics. Lack of description of the Y axis in the figures.

Response: The figure is redrawn using Statistics measurements, and Y axis is described.

Supplementary Figure I; Lack of a legend to the figure.

Response: Revised.

Supplementary Figure II; Lack of a legend to the figure. What do the numbers on the X axis mean? Lack of the Y axis unit.

Response: Revised.

Supplementary Figure III: illegible – needs to be improved.

Response: Revised and improved, enlarged to more legible and split into 3 parts.

Tables
Supplementary Table I: Please, check the names of the proteins; please, show the direction of the primer sequence.

Response: Names of the proteins, and primer sequence directions were corrected and revised.

Supplementary Table II: Please, check the title of the table, the names of the cultivars. Generally, the table needs to be improved.

Response: Revised.

Supplementary Table III: Generally, the table needs to be improved. ‘SI. No.’ – please explain what it means.

Response: Revised and improved.

References
Please, correct it; it should be prepared according to the manufacturer’s instructions.

Response: References were prepared according to the manufacturer’s instructions.

6. PLOS authors have the option to publish the peer review history of their article ([what does this mean?](https://nam03.safelinks.protection.outlook.com/?url=https%3A%2F%2Fjournals.plos.org%2Fplosone%2Fs%2Feditorial-and-peer-review-process%23loc-peer-review-history&data=02%7C01%7C%7Cbded8b049d264523e4b208d78dd0ed45%7C2526db3cd5034dfea0e60c41a20b52d2%7C0%7C1%7C637133800799451958&sdata=%2BGetucyGAj9FTDHJB2mexxecYPJ4i6kB6WhyYgelLPA%3D&reserved=0)). If published, this will include your full peer review and any attached files.

If you choose “no”, your identity will remain anonymous but your review may still be made public.

**Do you want your identity to be public for this peer review?** For information about this choice, including consent withdrawal, please see our [Privacy Policy](https://nam03.safelinks.protection.outlook.com/?url=https%3A%2F%2Fwww.plos.org%2Fprivacy-policy&data=02%7C01%7C%7Cbded8b049d264523e4b208d78dd0ed45%7C2526db3cd5034dfea0e60c41a20b52d2%7C0%7C1%7C637133800799461960&sdata=9Dv4HyB0j7MhOTnc4mipqhA2jsr5E9FcyN0B8D0CyRg%3D&reserved=0).

Reviewer #1: No

[NOTE: If reviewer comments were submitted as an attachment file, they will be attached to this email and accessible via the submission site. Please log into your account, locate the manuscript record, and check for the action link "View Attachments". If this link does not appear, there are no attachment files to be viewed.]

While revising your submission, please upload your figure files to the Preflight Analysis and Conversion Engine (PACE) digital diagnostic tool, [https://pacev2.apexcovantage.com/](https://nam03.safelinks.protection.outlook.com/?url=https%3A%2F%2Fpacev2.apexcovantage.com%2F&data=02%7C01%7C%7Cbded8b049d264523e4b208d78dd0ed45%7C2526db3cd5034dfea0e60c41a20b52d2%7C0%7C1%7C637133800799461960&sdata=UBSzD6WJxsGmL%2BxTfMZwl%2Fk6zba2sioz6iwmkyWwlSs%3D&reserved=0). PACE helps ensure that figures meet PLOS requirements. To use PACE, you must first register as a user. Registration is free. Then, login and navigate to the UPLOAD tab, where you will find detailed instructions on how to use the tool. If you encounter any issues or have any questions when using PACE, please email us at [figures@plos.org](mailto:figures@plos.org). Please note that Supporting Information files do not need this step.
